# One Health surveillance of multidrug-resistant diarrheagenic *Escherichia coli* in Northeast India

**DOI:** 10.3389/fmicb.2025.1667425

**Published:** 2025-10-13

**Authors:** Shivani Popli Goyal, Samaresh Das, Karma G. Dolma, Swagnik Roy, Tapan Kumar Dutta, Rajkumari Mandakini Devi, Megongusie Meru, Hosterson Kylla, W. Valarie Lyngdoh, Suranjana Chaliha Hazarika, Pallab Sarmah, Indira Sarangthem, Tapan Majumdar, Dilem Modi, Thandavarayan Ramamurthy, Madhuchhanda Das

**Affiliations:** ^1^Indian Council of Medical Research, New Delhi, India; ^2^Centre for Development of Advanced Computing, Kolkata, India; ^3^Department of Microbiology, Sikkim Manipal Institute of Medical Sciences, Sikkim Manipal University, Gangtok, Sikkim, India; ^4^Department of Microbiology, Zoram Medical College, Falkawn, Mizoram, India; ^5^College of Veterinary Sciences and Animal Husbandry, Central Agricultural University-Imphal, Aizawl, Mizoram, India; ^6^College of Veterinary Sciences and Animal Husbandry, Central Agricultural University-Imphal, Nagaland, India; ^7^Department of General Medicine, Christian Institute of Health Sciences and Research, Dimapur, Nagaland, India; ^8^State Disease Diagnostic Laboratory, Shillong, Meghalaya, India; ^9^North Eastern Indira Gandhi Regional Institute of Health and Medical Sciences, Shillong, Meghalaya, India; ^10^Gauhati Medical College and Hospital, Guwahati, India; ^11^ICMR- Regional Medical Research Centre, Dibrugarh, India; ^12^Institute of Bioresources and Sustainable Development (IBSD), Imphal, Manipur, India; ^13^Agartala Government Medical College, Agartala, Tripura, India; ^14^Bakin Pertin General Hospital, Pasighat, Arunachal Pradesh, India; ^15^ICMR-National Institute for Research in Bacterial Infections, Kolkata, India

**Keywords:** diarrheagenic *Escherichia coli*, antimicrobial resistance, One Health, multidrug resistance, surveillance

## Abstract

**Introduction:**

The emerging antimicrobial resistance (AMR) in diarrheagenic *Escherichia coli* (DEC) has become a critical public health concern worldwide. The present study provides a comprehensive surveillance report on the distribution and AMR profile of DEC in humans, animals and food across Northeast India using the “One Health” approach.

**Methods:**

Between October 2020 and December 2024, 8,149 stool specimens from hospitals, 20,691 market food and 2,094 animal samples were collected and screened for DEC. Identification of pathotypes and antimicrobial susceptibility testing were performed. Correlation and principal component analyses (PCA) were used to assess linkages of AMR profiles across the three sources.

**Results:**

DEC was detected in 61.2%, 20% and 28.2% of the samples, respectively, with predominance of enteropathogenic *E. coli*. Geographically, Sikkim exhibited the highest incidence of DEC in hospital samples (63.7%), followed by Mizoram (28.2%), while food surveillance suggested a higher prevalence in Mizoram (54%). AMR profiling revealed a high level of resistance against ampicillin (69.5%), azithromycin (68.4%) and cefoxitin (62%) in human isolates, with 75% classified as MDR/XDR. Food isolates also exhibited higher levels of resistance against ampicillin (83.2%), azithromycin (73.6%) and cephalosporins (46-70.9%), with MDR/XDR prevalence of 73.4%. Similarly, animal isolates showed higher level of resistance against ampicillin (71.6%), azithromycin (52%), cephalosporins (42.8-64%) and tetracycline (50.5%) with MDR/XDR prevalence of 88.2%. Emerging resistance against carbapenems in the three different sources has also been detected. Correlation analysis revealed a strong link between AMR patterns in humans and food (*r* = 0.95). Principal component analysis further corroborated the interaction between humans, food and animals DEC.

**Discussion:**

This study highlights the widespread burden of DEC and the alarming rise of MDR/XDR strains across humans, food and animals in Northeast India. The overlapping AMR patterns suggest shared reservoirs and transmission pathways, emphasizing the urgent need for stringent antimicrobial stewardship, a systematic surveillance system and improved hygiene practices to mitigate the spread of MDR-DEC.

## Graphical abstract

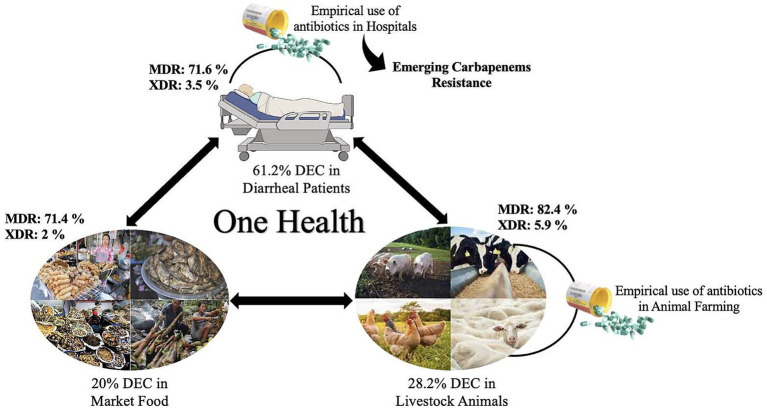

## 1 Introduction

Diarrheal diseases remain the third leading cause of morbidity and mortality worldwide, particularly among children under the age of five. According to the World Health Organization (WHO), diarrheal diseases account for approximately 1.7 billion cases globally, resulting in 0.495 million deaths every year, with a substantial burden in developing countries like India (WHO, 2024). Among the diverse pathogens responsible, *Escherichia coli* is the primary contributor to diarrheal illnesses. Despite its prevalence and impact, diarrheagenic *E. coli* (DEC) is often neglected and remains a critical public health concern (Abbasi et al., 2020). The alarming surge in antimicrobial resistant (AMR) DEC has further exacerbated this issue by reducing their effectiveness, which often leads to serious health complications.

DEC comprises a distinct group of five pathogenic strains of *E. coli*, including enteroinvasive *E. coli* (EIEC), enteropathogenic *E. coli* (EPEC), enterotoxigenic *E. coli* (ETEC), enteroaggregative *E. coli* (EAEC) and enterohemorrhagic *E. coli* (EHEC). Among them, EPEC, EAEC and ETEC are the predominant strains associated with infantile diarrhea in most developing countries (Bedane et al., 2024). The major source of transmission of such pathogens primarily includes contaminated food and water, as well as contact with animal reservoirs. Inadequate sanitation, poor hygiene practices and limited access to healthcare facilities further contribute to their prevalence. Various epidemiological studies have shown the association of contaminated fruits, vegetables, cookie dough, seafood, poultry and beef with DEC outbreaks (Wendel et al., 2009; Neil et al., 2012; Gaulin et al., 2015; Furukawa et al., 2018; Bedane et al., 2024). Moreover, the extensive usage of antimicrobials in animal farming and human health has led to the emergence of AMR, including multidrug resistance (MDR) and extensive drug resistance (XDR), across DEC (Heine et al., 2024; Tapia-Pastrana et al., 2024). Their interlinkage with several sources causes the rapid spread of AMR via horizontal gene transfer, enhancing the prevalence of drug-resistant DEC in diverse environments, and highlighting the indispensable need for a One Health approach (Heine et al., 2024).

In India, various national programs, such as Intensified Diarrhea Control Fortnight (IDCF), Swachh Bharat Abhiyan (Clean India mission) and the introduction of ‘Rotavirus vaccination’, have significantly reduced the mortality rate associated with diarrheal diseases (Moharana et al., 2019). Nevertheless, recent reports from Odisha, West Bengal and Northeast (NE) India suggested a surge in MDR DEC among animals and humans, emphasizing that diarrheal diseases remain a critical health concern in India (Moharana et al., 2019; Banerjee et al., 2022; Prasad et al., 2022).

The Indian Council of Medical Research (ICMR) initiated a task force project, ICMR-FoodNet, to conduct surveillance of foodborne pathogens, including DEC, aiming to map their prevalence AMR profiles and genetic diversity across NE India. Preliminary findings identified 18% of enteric bacteria as DEC, with 69% being EPEC. Similarly, hospital surveillance indicated that 71% of 184 strains isolated from diarrheal patients were DEC, with EPEC accounting for 79% of these cases (Das et al., 2024b). Additionally, several EPEC, EAEC and ETEC pathotypes from both market and hospital surveillance exhibited resistance to antimicrobials, such as ampicillin, azithromycin, cefotaxime, cefoxitin, ceftazidime and tetracycline (Das et al., 2024a). The lack of systematic reports on the prevalence of DEC pathotypes poses a challenge in developing targeted interventions and guiding national strategies to address this growing public health concern. Therefore, the present study aims to systematically evaluate the prevalence of drug-resistant DEC in diarrheal patients, animals and foods.

## 2 Methodology

### 2.1 Study design and sites

This community-based surveillance study was conducted between October 2020 and December 2024 under the ICMR-FoodNet taskforce project across NE India, with a population of approximately 45 million, where 70% of people depend on the agriculture sector for their livelihood (Ministry of Home Affairs | Government of India, 2025). The study constitutes an integrated network of medical (9) and veterinary (3) centers covering all eight NE states, i.e., Arunachal Pradesh, Assam, Manipur, Meghalaya, Mizoram, Nagaland, Sikkim and Tripura. Hospital and market surveillance were conducted in all 8 NE states, while animal surveillance was conducted only in Nagaland, Mizoram and Meghalaya. From each state, four districts were selected, representing the North, South, East and West zones of each state, with one hospital and one local market chosen for sample collection in each district.

#### 2.1.1 Ethical approval

The protocol was approved by the ICMR-Central Ethics Committee on Human Research (ICMR-CECHR) (Reference Number: CECHR 003/2023) and the Institutional Ethics Committees of each participating Institute. Written informed consent was obtained from all human participants, including food and animal handlers, before sample collection. Animal samples were collected in accordance with institutional ethical guidelines.

### 2.2 Sample collection

For hospital surveillance, 8,149 samples, such as stools/rectal swabs, were collected from diarrheal patients following the case definitions defined by IDSP guidelines (Das et al., 2024b). Market surveillance involved sample collection (20691) from various food categories, including dairy, fruits and vegetables, meat and meat products, fermented products and processed foods. Additionally, environmental samples like hand swabs, nasal swabs, kitchen clothes, surface swabs and utensils were also collected from street food vendors, shops and restaurants.

In animal surveillance, 2094 samples like feces, environmental samples from animal husbandries (water, soil and feed/fodder), animal handlers’ samples including nail bed scrapings, nasal swabs and swabs from the knives used for slaughtering were collected.

### 2.3 Laboratory investigation

#### 2.3.1 Isolation and identification of DEC

According to the methodology described by Standard Operating Procedures, samples were inoculated onto MacConkey and Cefixime Tellurite Sorbitol MacConkey (CT-SMAC) agar media to isolate *E. coli* (ICMR FoodNet SOP, 2022). Five isolated colonies were pooled from each sample and subjected to the DEC multiplex PCR assay. The primer sets used in the multiplex PCR are shown in Supplementary Table S1. If the pooled PCR is positive, individual colonies were tested for the specific DEC pathotype. The DEC isolates were sent to an external quality assurance (EQA) center, i.e., ICMR-National Institute for Research in Bacterial Infections (NIRBI), Kolkata, for confirmation. The confirmed DEC isolates were then tested for AMR.

#### 2.3.2 Antibiotic susceptibility testing

AST was carried out using the Kirby-Bauer disc diffusion method according to Clinical & Laboratory Standards Institute (CLSI) guidelines to assess the efficacy of 15 antimicrobials belonging to 12 different classes (CLSI M100, 2023). The tested antibiotic discs included ampicillin (AMP; 10 μg/disc), azithromycin (AZI, 15 μg/disc), cefoxitin (CXT, 30 μg/disc), cefepime (CPM, 30 μg/disc), cefotaxime (CTX, 30 μg/disc), ceftazidime (CTZ, 30 μg/disc), ceftriaxone (CTR, 30 μg/disc), chloramphenicol (CMP, 30 μg/disc), ciprofloxacin (CIP, 5 μg/disc), gentamicin (GEN, 10 μg/disc), imipenem (IMI, 10 μg/disc), meropenem (MEM, 10 μg/disc), nalidixic acid (NAL, 30 μg/disc), tetracycline (TET, 30 μg/disc) and trimethoprim-sulfamethoxazole (TMPSMX, 1.25/23.75 μg/disc). Briefly, using a sterile swab, Mueller–Hinton agar plates were inoculated, and specified antimicrobial discs were placed onto agar plates using sterile forceps. Following overnight incubation, zones of inhibition were measured, and results were interpreted as sensitive, intermediate or resistant according to the CLSI guidelines (2023), with *E. coli* ATCC 25922 being used as a quality control strain. If the isolate is non-susceptible to at least one antimicrobials in three or more classes, it is classified as MDR strain, while XDR strain is non-susceptible to at least one agent in all but two or fewer antimicrobial classes, i.e., one antimicrobial in 10 or more classes (Heine et al., 2024).

### 2.4 Data collection, management, and analysis

A secured web server was developed by the Centre for Development of Advanced Computing (CDAC), Kolkata, enabling all centers across eight NE states to register and input data digitally through a standardized electronic case report form (e-CRF). Internal quality control was monitored by ICMR-Regional Medical Research Centre (RMRC) Dibrugarh, which served as the site coordinating center, ensuring the accuracy and reliability of results. Conversely, EQA programs, laboratory training and maintenance of the pathogen repository were managed by ICMR-NIRBI, Kolkata.

Data analysis was done using MS Office Excel 365 and GraphPad Prism 9.5.0 for spreadsheet organization, statistical calculations (two-way ANOVA and chi-square (χ^2^) test) and advanced graphical visualization. The calculated *p*-values less than or equal to 0.05 were considered statistically significant. For subgroup analyses, Bonferroni correction was applied to adjust for multiple comparisons where statistical significance was observed. To study the relationship between different DEC pathotypes across humans, foods, and animals, Pearson’s correlation coefficient and principal component analysis (PCA) were applied using R Studio 4.3.1.

## 3 Results

### 3.1 Distribution of DEC pathotypes across humans, foods, and animals

**Figure 1:**
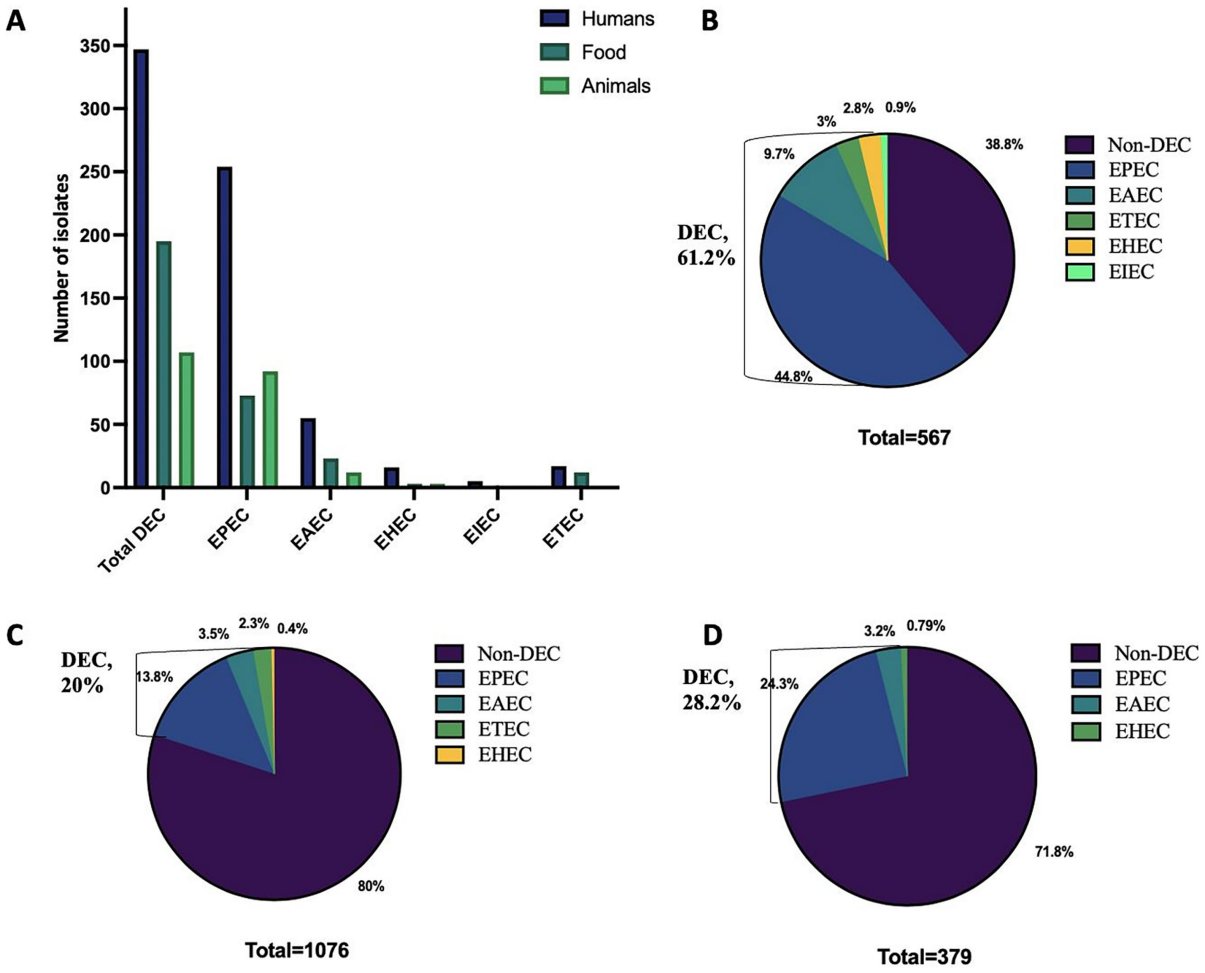
Distribution of various diarrhoeagenic *E. coli* (DEC) pathotypes in humans, animals and food across Northeast India. **(A)** Overall distribution. **(B)** Prevalence in humans. **(C)** Prevalence in food. **(D)** Prevalence in animals. EPEC, Enteropathogenic *E. coli*; EAEC, Enteroaggregative *E. coli*; ETEC, Enterotoxigenic *E. coli*; EHEC, Enterohaemorrhagic *E. coli*; EIEC, Enteroinvasive *E. coli.*

**Figure 2:**
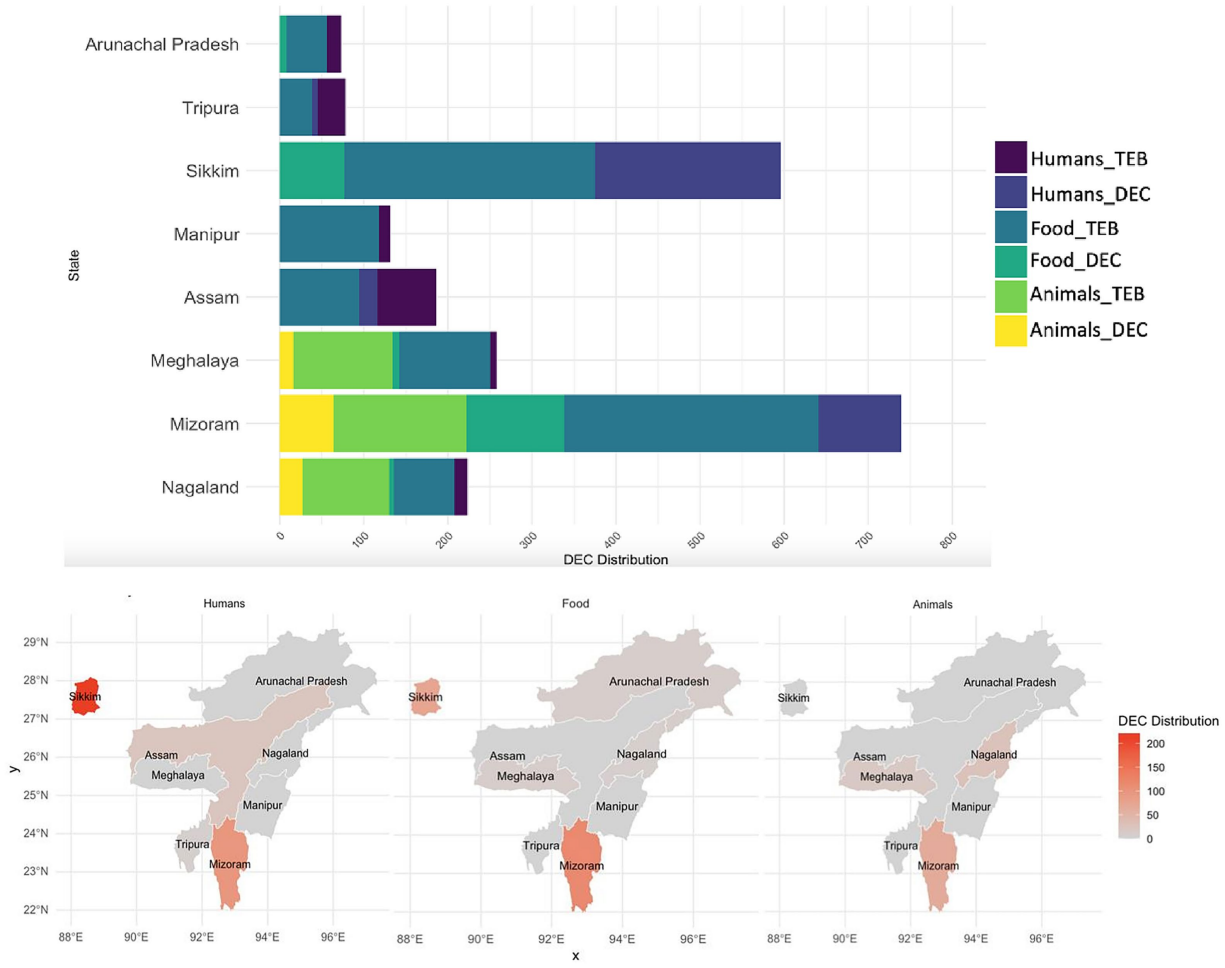
Distribution of total enteric bacteria (TEB) and diarrhoeagenic *E. coli* (DEC) in humans, food and animals across NE India. where, DEC Distribution represents number of isolates. Animal surveillance was conducted only in Nagaland, Mizoram and Meghalaya.

Among the 567 enteric bacteria isolated from 8,149 stool specimens, 347 isolates were identified as DEC, accounting for 61.2% of the enteric bacteria, with a predominance of EPEC (73.2%), followed by EAEC (15.8%) (Figures 1A,B). Temporal distribution of DEC isolates revealed a progressive increase in overall hospital case numbers between 2021 and 2024 (Supplementary Figure S1a). From 2022 onward, DEC increased from 43 to >200 isolates by 2024, with EPEC showing the steepest rise from 27 to >150, while EHEC, EIEC, and ETEC remained low. DEC cases were primarily prevalent in the age group of 6 to 15 years (5.19%), but this variation was insignificant (χ^2^ = 4.54, *p* = 0.34). Similarly, no significant gender-wise difference was observed in DEC prevalence (female: 4.3%, male: 4.2%; χ^2^ = 0.003, *p* = 0.95) with a female-to-male ratio of 1.03:1. A higher proportion of positives was detected among inpatients than outpatients (4.75% vs. 0.53%; χ^2^ = 34.41, *p* < 0.001). Analysis of clinical features revealed that vomiting (5.68%; OR 1.92, 95% CI 1.54–2.40, *p* < 0.001), nausea (5.70%; OR 1.50, 95% CI 1.18–1.91, *p* = 0.001) and blood in stool (1.74%; OR 0.38, 95% CI 0.20–0.75, *p* = 0.005) were significantly associated with DEC positivity. In contrast, fever (4.35%; OR 1.05, 95% CI 0.84–1.30), diarrhea (4.32%; OR 1.89, 95% CI 0.83–4.28) and abdominal pain (4.24%; OR 0.99, 95% CI 0.80–1.23) showed no significant association with DEC prevalence (*p* > 0.05). All patients were eventually cured (Table 1). Geographically, a higher incidence of DEC was detected in Sikkim (221/347; 63.7%) followed by Mizoram (98/347; 28.2%) (*p* ≤ 0.05) (Figure 2). However, market surveillance revealed a higher prevalence of DEC in Mizoram (116/215; 54%) followed by Sikkim (77/215; 35.8%) from food, water and environment (*p* ≤ 0.05). DEC accounted for 20% of the total enteric bacteria (215/1076) in market samples, with EPEC being the major pathotype (68.8%) (*p* ≤ 0.05) (Figure 1C). A progressive increase in overall DEC was observed between 2021 and 2024, with EPEC showing the steepest rise from 28 to 91 isolates, while EAEC, ETEC and EHEC remained low (Supplementary Figure S1b). Raw foods (51.2%; 110/215), particularly fermented/processed food (2.48%) as well as dough and batter (2.84%), were found to be highly contaminated with DEC compared to cooked foods (39.5%; 85/215) (χ^2^ = 74.8, *p* < 0.001). Nevertheless, the overall DEC detection rate was slightly higher in cooked foods (1.2%) than in raw foods (1.01%), possibly due to larger sample sizes. A high level of DEC was detected in cooked foods like dairy (1.8%) and refrigerated foods (2.05%) (χ^2^ = 6.74, *p* = 0.15). DEC was also detected in 1.41% of water samples, notably from drinking and tap sources. Similarly, in the case of environmental samples, the overall DEC detection rate was 0.54% (10/1846), with isolates recovered from kitchen cloths (3/10) and surface swabs (3/10).

**Table 1:** Prevalence of total enteric bacteria (TEB) and diarrheagenic *E. coli* (DEC) positivity in human, food, water, and animal sources across Northeast India.

Source	Group	Sample	TEB positivity	DEC positivity	DEC n %	χ^2^/odd ratio (95% CI) (*p*-value)
**Human stools**	**Age**					4.54 (*p* = 0.34)
	0–5	1,498	86	58	3.87	
	6–15	1,001	77	52	5.19	
	16–30	2,286	169	92	4.02	
	31–45	1,689	123	80	4.74	
	≥46–60	1,675	112	65	3.88	
	**Total**	**8,149**	**567**	**347**	**4.26**	
	**Gender**					0.003 (*p* = 0.95)
	Female	4,121	282	176	4.27	
	Male	4,028	285	171	4.24	
	**Total**	**8,149**	**567**	**347**	**4.26**	
	**Hospitalization**					34.41 (*p* < 0.001)
	Outpatient	942	30	5	0.53	
	Inpatient	7,207	537	342	4.75	
	**Total**	**8,149**	**567**	**347**	**4.26**	
	**Clinical features**					
	Fever	3,976	331	173	4.35	1.05 (0.84–1.30) (*p* < 0.001)
	Vomiting	3,770	323	214	5.68	1.92 (1.54–2.40) (*p* < 0.001)
	Diarrhoea	7,892	505	341	4.32	1.89 (0.83–4.28) *p* = 0.163
	Nausea	1701	179	97	5.70	1.50 (1.18–1.91) *p* = 0.001
	Blood in stool	516	44	9	1.74	0.38 (0.20–0.75) *p* = 0.005
	Abdominal pain	3,611	285	153	4.24	0.99 (0.80–1.23) *p* = 0.977
	**Outcome**					
	Cured	8,149	567	347	4.26	
**Market**	**Cooked food**					6.74 (*p* = 0.15)
	Dairy	1,242	107	22	1.77	
	Meat	1857	151	18	0.97	
	Refrigerated	292	15	6	2.05	
	Rice, flour, pulses	2,180	103	24	1.10	
	Vegetables	1,503	55	15	0.99	
	**Total**	**7,074**	**431**	**85**	**1.2**	
	**Raw food**					74.8 (*p* < 0.001)
	Dough and batter	528	34	15	2.84	
	Fermented/processed/preserved	646	42	16	2.48	
	Fruits, vegetables, and salads	3,585	145	16	0.45	
	Milk	368	11	2	0.54	
	Raw/Dried Meat	3,153	209	54	1.71	
	Raw/dry fish	2,539	118	6	0.24	
	Spices	107	3	1	0.93	
	**Total**	**10,926**	**562**	**110**	**1.01**	
	**Water**	711	46	10	1.41	
	**Environment**	1846	37	10	0.54	
**Animal farms**	**Animal food products**					7.84 (*p* = 0.09)
	Eggs	189	24	10	5.29	
	Meat (chicken, porcine, and bovine)	762	126	17	2.23	
	Meat products	17	6	0	0	
	Milk	218	27	10	4.59	
	Milk products	21	1	0	0	
	**Total**	**1,207**	**184**	**37**	3.06	
					
	**Animal handler/butcher**					2.39 (*p* = 0.30)
	Nail bed scrap	64	6	0	0	
	Nasal swab	2	0	0	0	
	Knife swab	113	14	4	3.54	
	Total	179	20	4	2.23	
	**Animal living environment**					2.42 (*p* = 0.30)
	Feed/fodder	117	5	0	0	
	Soil	75	8	1	1.33	
	Water	64	3	0	0	
	**Total**	**256**	**16**	**1**	**0.39**	
					
	**Animal sample**					1.88 (*p* = 0.60)
	Feces	1,295	142	57	4.40	
	Intestinal content from carcass	52	4	2	3.85	
	Other discharges	39	3	1	2.56	
	Rectal/cloacal swab	66	10	5	7.58	
	**Total**	**1,452**	**159**	**65**	**4.48**	

Animal surveillance also exhibited a notable incidence of DEC up to 28.2% (107/379) of the total enteric bacteria isolated, with significantly higher detection in Mizoram (59.8%; 64/107) (*p* ≤ 0.05). A high level of DEC was detected in animal samples (60.74%; 65/107), particularly stools (57/107), with a predominance of EPEC up to 86.15%. Similarly, DEC detected in animal-derived food products (34.6%; 37/107) was primarily EPEC (86.4%), with the higher detection compared to chicken and porcine meat samples (17/107), highlighting the zoonotic potential of animal sources (Figure 1C). However, the detection rate was highest in eggs (5.29%) and milk (4.59%), compared to meat (2.23%) (χ^2^ = 7.84, *p* = 0.09).

### 3.2 Antimicrobial resistance profiling

**Figure 3:**
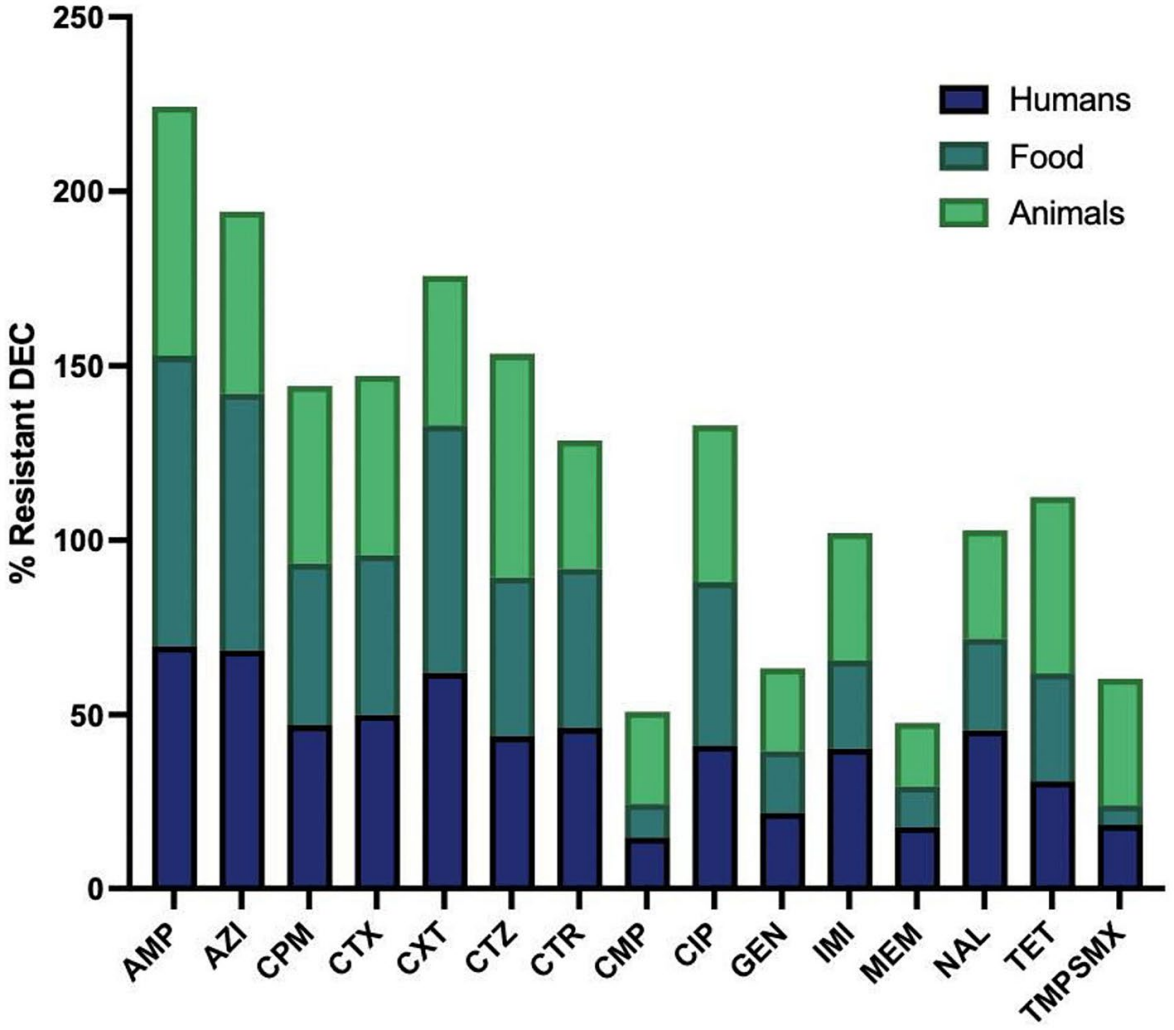
Resistant rates of diarrhoeagenic *E. coli* (DEC) against different antimicrobials in humans, animals and food. Ampicillin, AMP: *n* = 335, 196, 102; Azithromycin, AZI: *n* = 329, 178, 67; Cefepime, CPM: *n* = 340, 199, 102; Cefotaxime, CTX: *n* = 338, 197, 101; Cefoxitin, CXT: *n* = 253, 103, 56; Ceftazidime, CTZ: *n* = 335, 198, 100; Ceftriaxone, CTR: *n* = 316, 175, 98; Chloramphenicol, CMP: *n* = 332, 196, 102; Ciprofloxacin, CIP: *n* = 308, 184, 100; Gentamicin, GEN: *n* = 337, 199, 101; Imipenem, IMI: *n* = 318, 190, 71; Meropenem, MEM: *n* = 324, 199, 93; Nalidixic acid, NAL: *n* = 226, 103, 87; Tetracycline, TET: *n* = 249, 110, 91; Trimethoprim-sulfamethoxazole, TMPSMX: *n* = 206, 125, 102. Where n is the number of isolates tested for the antibiotic susceptibility test.

**Figure 4:**
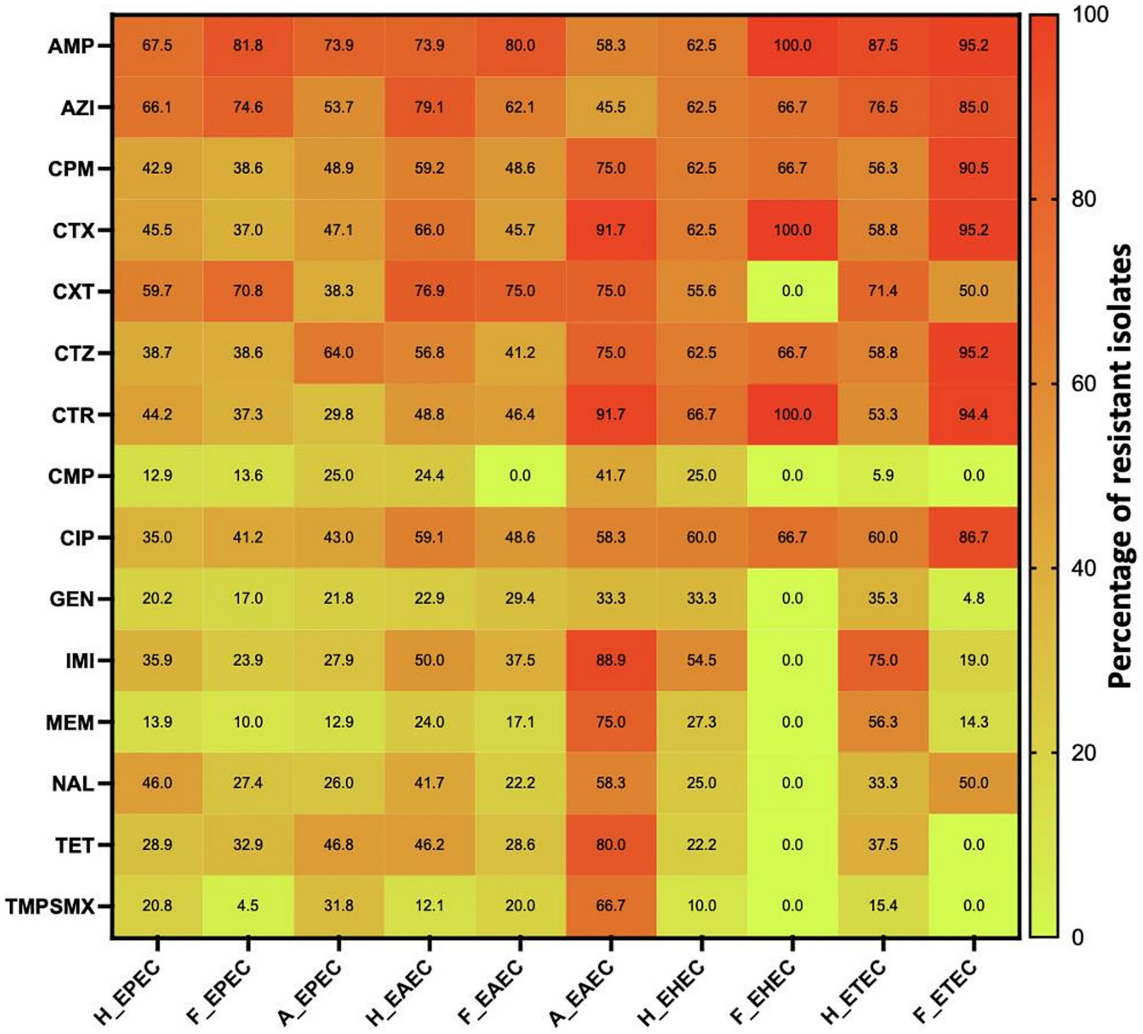
AMR pattern of various pathotypes of diarrhoeagenic *E. coli* pathotypes against different antimicrobials in humans, animals and food. Number of isolates tested is variable. H_EPEC, H_EAEC, H_EHEC & H_ETEC are DEC strains in humans; F_EPEC, F_EAEC, F_EHEC & F_ETEC are DEC strains; A_EPEC & A_EAEC are DEC strains in animals. AMP, Ampicillin; AZI, Azithromycin; CPM, Cefepime; CTX, Cefotaxime; CXT, Cefoxitin; CTZ, Ceftazidime; CTR, Ceftriaxone; CMP, Chloramphenicol; CIP, Ciprofloxacin; GEN, Gentamicin; IMI, Imipenem; MEM, Meropenem; NAL, Nalidixic acid; TET, Tetracycline; TMPSMX, Trimethoprim-sulfamethoxazole.

Figures 3, 4 illustrate the overall AMR rates and DEC pathotype-specific resistance patterns, respectively, from different sources. A total of 206 to 341 DEC isolates from hospital surveillance were processed for AST, revealing higher resistance across all five DEC pathotypes, particularly against ampicillin, azithromycin, cefotaxime, ceftazidime, imipenem and meropenem. As shown in Supplementary Table S2, most of the isolates exhibited resistance to ≥5 tested antimicrobials. The most common antibiogram detected was for 11 antimicrobials, i.e., AMP-AZI-CIP-CPM-CTR-CTX-CTZ-CXT-GEN-NAL-TET, with a frequency of 18/341 isolates. Notably, 71.6% (244/341) of the tested isolates were classified as MDR, while 3.5% (12/341) exhibited an XDR profile (Supplementary Table S4). The predominant pathotype, EPEC, showed the highest AMR prevalence, with 71.3% (174/244) of MDR isolates and 75% (9/12) of XDR. EPEC showed resistance against all the tested antimicrobial classes, with resistance rates ranging from 13 to 68%. The maximum resistance was observed against ampicillin at 67.5% (170/252), azithromycin at 66.1% (164/248) and cefoxitin at 60% (123/206). For EAEC, resistance was observed between 12 and 79%, with a higher level of resistance against ampicillin (73.9%; 34/46), azithromycin (79%; 34/43) and cefoxitin (65.9%; 31/47). Similarly, ETEC exhibited the highest resistance against ampicillin (14/16), azithromycin (13/17) and imipenem (12/16), while EHEC isolates were more resistant to ampicillin (10/16), azithromycin (10/16), cefepime (10/16), cefotaxime (10/16), ceftazidime (10/16), ceftriaxone (10/15) and ciprofloxacin (9/15). Among five EIEC isolates, four were found to be resistant against azithromycin, cefoxitin, ceftazidime and ciprofloxacin, while all five exhibited resistance to ampicillin.

Under market surveillance, the number of DEC tested for AST ranged from 103 to 199, with prevalent resistance to ampicillin, ciprofloxacin and tetracycline. Supplementary Table S3 depicts the most frequently detected AMR patterns, i.e., AMP-AZI-CPM-CTR-CTX-CTZ (10.1%; 20/199) and AMP-AZI-CIP-CPM-CTR-CTX-CTZ (8.5%; 17/199). A significant proportion of DEC was classified as MDR (71.4%; 142/199), while 2.01% (4/199) were identified as XDR (Supplementary Table S5). EPEC exhibited the maximum resistance to ampicillin, with 81.7% of isolates (112/137) being resistant, followed by azithromycin at 74.6% (94/126) and cefoxitin at 70.8% (51/72). Resistance was also observed against ciprofloxacin (41.2%; 54/131), cefepime (38.6%; 54/140), ceftazidime (38.6%; 54/140) and cefotaxime (36.9%; 51/138). EAEC also demonstrated higher resistance against ampicillin (80%; 28/35), azithromycin (62.1%; 18/29), cefepime (48.6%; 17/35), cefotaxime (45.7%; 16/35), cefoxitin (75%; 21/28) and ciprofloxacin (48.6%; 17/35). Similarly, ETEC isolates were found to be highly resistant to ampicillin (20/21), azithromycin (17/20), cefepime (19/21), cefotaxime (20/21), ceftazidime (20/21) and ceftriaxone (17/ 18), while all three EHEC were resistant to ampicillin, cefotaxime and ceftriaxone.

In animal surveillance, most of the DEC showed resistance to ≥5 antimicrobials, with widespread resistance to ampicillin, ciprofloxacin and tetracycline. Notably, 0.03% (3/102) of isolates exhibited resistance to all 15 selected antimicrobials. A significant proportion, 82.4% (84/102) of isolates were classified as MDR, while 5.9% (6/102) exhibited an XDR profile (Supplementary Table S6). Among EPEC, resistance was detected against ampicillin (73.9%; 65/88), cefepime (48.9%; 43/88), cefotaxime (47%; 41/87), ceftazidime (63.9%; 55/86), ciprofloxacin (43%; 37/86) and tetracycline (46.8%; 37/79). Similarly, EAEC demonstrated high resistance, with eight or more isolates being observed to be resistant to cefepime, cefotaxime, ceftazidime, ceftriaxone, imipenem, tetracycline and trimethoprim-sulfamethoxazole.

### 3.3 Correlation and principal component analysis

**Figure 5:**
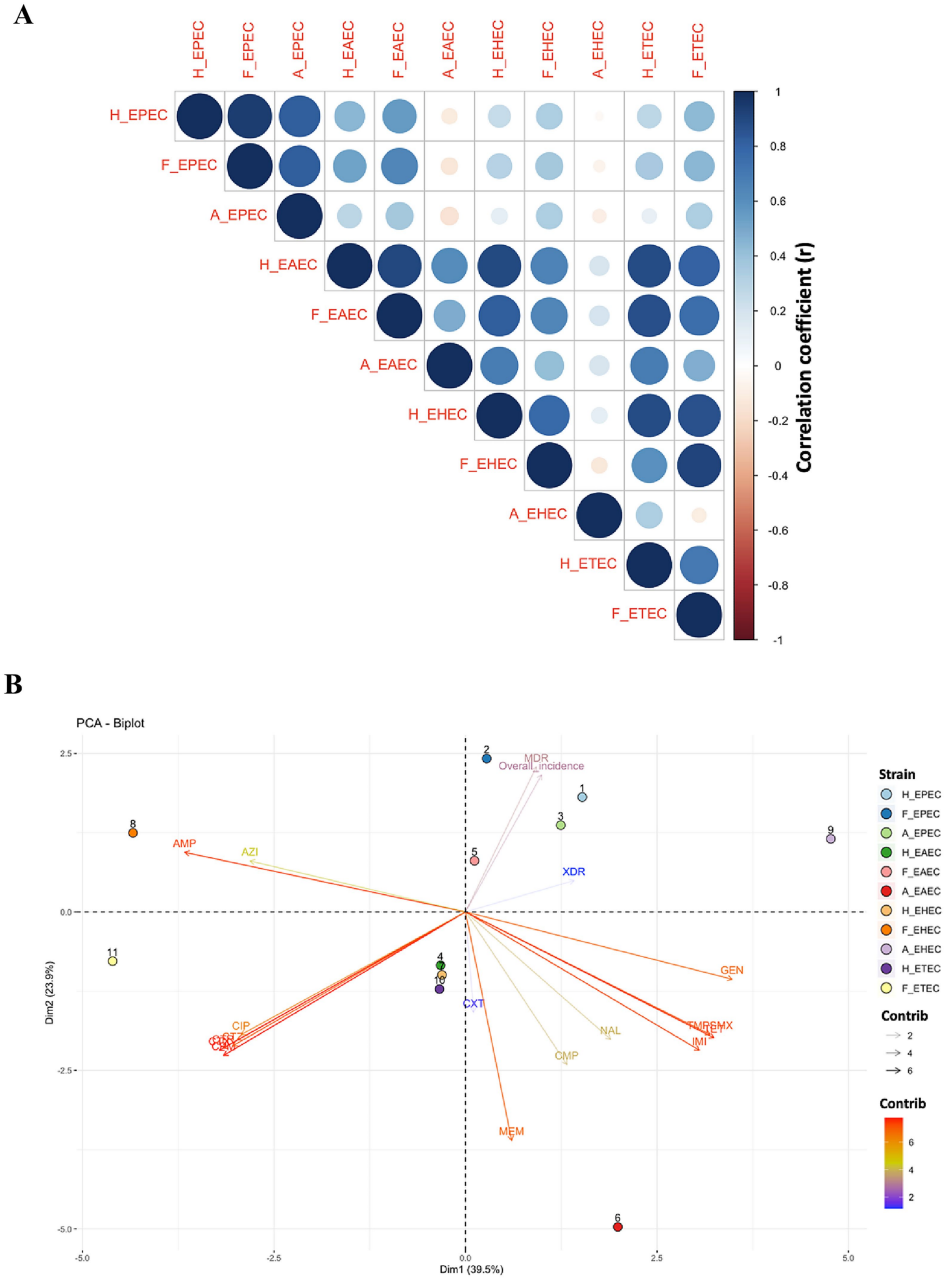
Interaction between different pathotypes of diarrhoeagenic *E. coli* in humans, animals and food. **(A)** Corrplot: color gradient and circle size represent correlation coefficient between different DEC pathotypes isolated from humans, food, and animals. **(B)** PCA Biplot. H_EPEC, H_EAEC, H_EHEC and H_ETEC are DEC strains in humans; F_EPEC, F_EAEC, F_EHEC and F_ETEC are DEC strains; A_EPEC and A_EAEC are DEC strains in animals. AMP, Ampicillin; AZI, Azithromycin; CPM, Cefepime; CTX, Cefotaxime; CXT, Cefoxitin; CTZ, Ceftazidime; CTR, Ceftriaxone; CMP, Chloramphenicol; CIP, Ciprofloxacin; GEN, Gentamicin; IMI, Imipenem; MEM, Meropenem; NAL, Nalidixic acid; TET, Tetracycline; TMPSMX, Trimethoprim-sulfamethoxazole.

To further assess the interaction between different DEC in humans, foods and animals, the association between AMR, MDR, XDR and overall incidence was estimated using Pearson’s correlation coefficient and PCA analysis. Correlation analysis (Figure 5A) revealed a stronger association between AMR in human and food isolates (*r* = 0.95, *p* < 0.001), compared to human vs. animal (*r* = 0.72, *p* < 0.01) and food vs. animal isolates (*r* = 0.76, *p* < 0.001), indicating a critical link between food and human AMR patterns. Strong positive correlations were also observed for specific pathotypes, including H_EPEC and F_EPEC (*r* = 0.95), H_EAEC and F_EAEC (*r* = 0.91), H_EHEC and F_EHEC (*r* = 0.78), as well as H_ETEC and F_ETEC (*r* = 0.71), suggesting shared resistance patterns, potentially influenced by common selective pressures. Moreover, the correlation between H_EPEC and A_EPEC (*r* = 0.82), as well as H_EAEC and A_EAEC (*r* = 0.61), suggested possible zoonotic associations, though causality remains unconfirmed.

PCA biplot (Figure 5B) further substantiated the interaction between various *E. coli* pathotypes in three different sources. The biplot exhibited a total variability of 63.4%, with Dim1 accounting for 39.5% of the variance and Dim2 contributing 23.9%, suggesting distinct strain clustering and resistance patterns to different antimicrobials. AMP and AZI, situated in quadrant II, exhibited a strong correlation, indicating frequent co-occurrence. Similarly, cephalosporins (CTR, CTX, CTZ, CPM) also showed high association and clustering in quadrant III. The close proximity of TET and TMPSMX in quadrant IV also indicated possible co-selection. Different DEC displayed distinct spatial distributions owing to pathotype-specific resistance patterns. Notably, H_EPEC, F_EPEC, and A_EPEC were clustered in quadrant I, suggesting shared selection pressure of resistance across these sources. Their position also coincides with MDR and overall incidence, corroborating their predominance. The overall scattering of different DEC pathotypes across four quadrants highlighted the influence of bacterial environment, pathotype-specific adaptations and antibiotic exposure on variations in resistance selection pressure.

## 4 Discussion

Northeast India, with its unique agricultural practices and traditional ethnic foods, including fermented products like fish, soybeans, bamboo shoots and vegetables, is predisposed to diarrheal illnesses due to their long-term storage and secondary contamination. Among the diverse enteric pathogens, DEC is a critical contributor to overall diarrheal cases (Das et al., 2024c). However, absence of a systematic surveillance system makes it challenging to monitor their prevailing trends and address the rising MDR pathotypes. Furthermore, no intensive study has been made interlinking DEC occurrence in humans, animals and foods. To address this gap, the present investigation evaluated the occurrence of drug-resistant DEC in humans, animals and foods, and correlated their AMR profile in these domains, adopting a One Health approach to combat this growing public health challenge.

In the current study, a higher incidence of DEC (61.2%) was observed in diarrheal patients compared to other enteric bacteria, suggesting it to be a primary contributor to the total diarrheal cases. This observation is consistent with the results of Ndjangangoye et al. (2023) and Sharif et al. (2023), who also reported a higher prevalence of *E. coli* among diarrheal children in Central Africa and Bangladesh, respectively, compared to other enteric bacteria. The present study also showed EPEC (44.8%) as the predominant DEC pathotype detected in diarrheal patients, followed by EAEC (9.7%) and ETEC (3%), which is in accordance with the recent studies by Singh et al. (2022) and Tapia-Pastrana et al. (2024), from East Delhi, India (18%) and Southwest Mexico (51%), respectively. Conversely, another study from NE India observed a higher prevalence of EAEC, followed by EPEC, in a tertiary care center among children under 18 years (Prasad et al., 2022). This pathotype dominance pattern is probably due to the inclusion of diverse age groups in the present study. Nevertheless, EPEC, EAEC and ETEC are considered to be major causes of diarrhea worldwide (Snehaa et al., 2021). The present study also detected a similar trend with respect to EPEC dominance in both animal (13.8%) and market (24.3%) surveillance, suggesting overlapping reservoirs between humans, animals and foods. However, compared to hospital surveillance, the detection rate of total DEC remained lower in animal (28.2%) and market (20%) foods compared to other enteric bacteria.

A plethora of studies have revealed the emerging concerns related to the rising AMR among DEC (Abbasi et al., 2020; Snehaa et al., 2021; Prasad et al., 2022; Ndjangangoye et al., 2023; Heine et al., 2024; Tapia-Pastrana et al., 2024). Recently, a report from South Africa revealed that 164 out of 166 DEC from diarrheal children were MDR, with 4.9% of DEC being XDR and 92.2% being extended-spectrum *β*-lactamase (ESBL) producers (Heine et al., 2024). Similarly, a study from Nigeria observed a 63% prevalence of DEC strains in 230 children with diarrhea, where 40% of DEC were identified as MDR and XDR (Tapia-Pastrana et al., 2024). Moreover, MDR DEC have been detected in Bengal goats and piglets in India, suggesting a potential risk of zoonotic transmission (Puii et al., 2019; Banerjee et al., 2022). The findings of this study also highlight alarming rates of AMR among DEC from humans, animals and foods. In humans, 75% of them were found to be MDR and XDR, while market and animal surveillance exhibited the occurrence of 73.4 and 88.2%, respectively, underscoring the urgent need for enhanced monitoring and antimicrobial stewardship programs. Moreover, the correlation matrix derived from Pearson’s correlation coefficients (corrplot) revealed a strong positive association of AMR between humans and food, suggesting overlapping reservoirs and shared selection pressures. While these findings highlight potential epidemiological linkages. However, correlation alone cannot establish a strong link between DEC and suspected sources. Future genomic studies, including molecular typing and whole-genome sequencing, are warranted to confirm resistance mechanisms and strain clonality to strengthen the hypothesis of inter-source transmission pathways of DEC.

Across all three main sources, most DEC, particularly EPEC and EAEC, exhibited a higher level of resistance against ampicillin, azithromycin, cefepime, cefotaxime, cefoxitin, ceftazidime and ciprofloxacin, which is in agreement with previous reports (Chellapandi et al., 2017; Moharana et al., 2019; Puii et al., 2019; Banerjee et al., 2022; Prasad et al., 2022). A majority of the isolates were found to be resistant to β-lactam antimicrobials, such as ampicillin and cephalosporins, and phenotypically considered to be ESBL producers. However, a key limitation of the present study is the lack of genomic characterization to identify specific ESBL genes, such as *bla*_SH*V*_*, bla*_CTX-M_ and *bla*_TEM_. Future genomic studies are essential to elucidate the underlying resistance mechanisms and confirm the presence of specific AMR determinants. Notably, a previous study from NE India has reported the rising incidence of ESBL genes, particularly the *bla*_SHV_ gene, among enteric bacteria (Puii et al., 2019). The extensive usage of these antimicrobials in human and veterinary medicine, particularly swine and poultry production, is widely linked to the emergence of ESBL-producing DEC. Moreover, animals and their meat are often considered reservoirs of ESBL-producing *E. coli* (Bergšpica et al., 2020; Kim et al., 2020; Ahmed et al., 2023). In the present study, 26.7% of human isolates, 25.8% of animal isolates, and 18.8% of food isolates exhibited carbapenem resistance, which is often used as a last resort for treating ESBL-resistant infections (Sheu et al., 2019). Interestingly, carbapenems are not employed for veterinary use in NE India, suggesting the possible dissemination of resistance among DEC pathotypes from human clinical use or transfer of resistance determinants through environmental or food-chain pathways (Puii et al., 2019). The Corrplot and PCA biplot indicated a strong association of AMP-AZI, cephalosporins and TET-TMPSMX, suggesting possible co-selection due to the occurrence of AMR encoding genes on the same mobile genetic elements like plasmids (Ozawa et al., 2023). Moreover, the antibiotic pattern, AMP-AZI-CIP-CPM-CTR-CTX-CTZ-CXT-GEN-NAL-TET, was recurrent in hospital surveillance across multiple DEC pathotypes, while AMP-AZI-CPM-CTR-CTX-CTZ was recurrent in market surveillance, suggesting either possible transmission of these AMR patterns among different DEC or potential co-selection pressures (Peirano and Pitout, 2019). However, molecular typing is needed to confirm the clonal spread or dominance of a high-risk lineage. High resistance rates were also observed for quinolones, fluoroquinolones and tetracycline, which are widely employed in the livestock sector. These findings were consistent with the observations of Kim et al. (2020), who also reported strong resistance to quinolones (including fluoroquinolones), penicillins and tetracyclines in poultry meats (Kim et al., 2020).

Overall, the above findings suggest a high AMR prevalence in several DEC pathotypes across all three settings, highlighting the interconnected nature of AMR transmission at the human–animal–food interface.

### 4.1 Limitations of the study

While this study offers significant strengths, including comprehensive surveillance data collected through an integrated network of veterinary and medical centers across all North Eastern states and inclusion of DEC isolates from human, animal, and food sources, it also has a few limitations, including a cross-sectional design, variation in sample availability across sites, difference in sources and pathotypes, absence of antimicrobial usage (AMU) data and the lack of genomic analysis to confirm resistance mechanisms or transmission pathways. Future investigations involving whole-genome sequencing, multi-locus sequence typing or single-nucleotide polymorphism-based phylogenetic analysis, along with AMU data, are warranted to genetically characterize AMR in DEC and to confirm potential epidemiological linkages.

Despite these constraints, the study highlights the critical need for stringent antimicrobial stewardship in healthcare and animal husbandry, improved hygiene practices and systematic surveillance programs to further curb the spread of resistant DEC strains.

## 5 Conclusion

The present study is the first comprehensive report providing evidence for the widespread occurrence of DEC pathotypes in humans, foods and animals across NE India. The findings of this study highlight a critical public health challenge, which requires immediate attention to reduce diarrheal prevalence, treatment failures and complications. The emerging trends of resistance to carbapenems underscore an urgent need for judicious use of this drug in healthcare and for vigilance to prevent further cross-sectoral spread. To address this issue, immediate interventions, including policy reforms on over-the-counter antibiotic sales, inclusion of food handlers in AMR awareness and hygiene training initiatives, improved diagnostic methods, including targeted sequencing to detect resistance markers, identification of mutational and chromosomal/plasmid for antimicrobial resistance, and establishment of a robust surveillance system, are essential to limit the further spread of AMR and ensure the efficacy of current antimicrobial treatments.

## Data Availability

The original contributions presented in the study are included in the article/Supplementary material, further inquiries can be directed to the corresponding author.
