# Supplementary material for: One Health surveillance of multidrug-resistant diarrheagenic Escherichia coli in Northeast India
Source: Front Microbiol. 2025 Oct 13;16:1667425. doi: 10.3389/fmicb.2025.1667425 (PMC12554735; doi:10.3389/fmicb.2025.1667425)
Supplement: Supplementary file 1 [file Table_1.docx]

***Supplementary Material***

**Table S1. List of primers for detection of DEC pathotypes**

| **Genes** | **Forward** | **Reverse** |
| --- | --- | --- |
| **DEC multiplex PCR** | | |
| **EHEC** | | |
| *stx1* | 5’-CAG TTA ATG TGG TGG CGA AGG-3’ | 5’-CAC CAG ACA ATG TAA CCG CTG-3’ |
| *stx2* | 5’-ATC CTA TTC CCG GGA GTT TAC G-3’ | 5’-GCG TCA TCG TAT ACA CAG GAG C-3’ |
| **EPEC** | | |
| *eae* | 5’-TCA ATG CAG TTC CGT TAT CAG TT-3’ | 5’-GTA AAG TCC GTT ACC CCA ACC TG-3’ |
| *bfp* | 5’-GGA AGT CAA ATT CAT GGG GGT AT-3’ | 5’-GGA ATC AGA CGC AGA CTG GTA GT-3’ |
| **ETEC** | | |
| *elt* | 5’-GCA CAC GGA GCT CCT CAG TC-3’ | 5’-TCC TTC ATC CTT TCA ATG GCT TT-3’ |
| *est* | 5’-AAA GGA GAG CTT CGT CAC ATT TT-3’ | 5’-AAT GTC CGT CTT GCG TTA GGA C-3’ |
| **EIEC** | | |
| *virF* | 5’-AGC TCA GGC AAT GAA ACT TTG AC-3’ | 5’-TGG GCT TGA TAT TCC GAT AAG TC-3’ |
| *ipaH* | 5’-CTC GGC ACG TTT TAA TAG TCT GG-3’ | 5’-GTG GAG AGC TGA AGT TTC TCT GC-3’ |
| **DAEC** | | |
| *daaE* | 5’-GAA CGT TGG TTA ATG TGG GGT AA-3’ | 5’-TAT TCA CCG GTC GGT TAT CAG T-3’ |
| **EAEC** | | |
| *aafII* | 5’-CAC AGG CAA CTG AAA TAA GTC TGG-3’ | 5’-ATT CCC ATG ATG TCA AGC ACT TC-3’ |
| *astA* | 5’-TGCCATCAACACAGTATATCCG-3’ | 5’-ACGGCTTTGTAGTCCTTCCAT-3’ |
| **EAEC multiplex PCR** | | |
| *aap* | 5’-CTT GGG TAT CAG CCT GAA TG-3’ | 5’-AAC CCA TTC GGT TAG AGC AC-3’ |
| *aggR* | 5’-CTA ATT GTA CAA TCG ATG TA-3’ | 5’-AGA GTC CAT CTC TTT GAT AAG-3’ |
| *AA probe* | 5’-CTG GCG AAA GAC TGT ATC AT-3’ | 5’-CAA TGT ATA GAA ATC CGC TGT T-3’ |
