# Supplementary material for: One Health surveillance of multidrug-resistant diarrheagenic Escherichia coli in Northeast India
Source: Front Microbiol. 2025 Oct 13;16:1667425. doi: 10.3389/fmicb.2025.1667425 (PMC12554735; doi:10.3389/fmicb.2025.1667425)
Supplement: Supplementary file 2 [file Table_2.docx]

***Supplementary Material***

**Table S2. Antibiogram of diarrheagenic *Escherichia coli* isolated from hospital surveillance***

| **Number of Antimicrobials** | **Resistance Pattern** | **Number Observed** |
| --- | --- | --- |
| 3 | AMP-AZI-CXT | 6 |
| 4 | AMP-AZI-IMI-MEM  AMP-AZI-IMI-MEM  AMP-CXT-IMI-TET  AZI-CTX-CTZ-NAL | 6  6  6  5 |
| 6 | AMP-AZI-CIP-CPM-CTX-CTZ | 8 |
| 8 | AMP-AZI-CIP-CPM-CTX-CTZ-IMI-MEM  AMP-CIP-CPM-CTX-CTZ-IMI-MEM-TMPSMX  AMP-AZI-CPM-CTR-CTX-CXT-IMI-TMPSMX | 7  6  11 |
| 11 | AMP-AZI-CIP-CPM-CTR-CTX-CTZ-CXT-GEN-NAL-TET | 18 |

***Note**: Only resistance patterns observed in five or more isolates with similar resistance profiles are presented. For animal isolates, no resistance pattern met this threshold.
