# Supplementary material for: One Health surveillance of multidrug-resistant diarrheagenic Escherichia coli in Northeast India
Source: Front Microbiol. 2025 Oct 13;16:1667425. doi: 10.3389/fmicb.2025.1667425 (PMC12554735; doi:10.3389/fmicb.2025.1667425)
Supplement: Supplementary file 3 [file Table_3.docx]

***Supplementary Material***

**Table S3. Antibiogram of diarrheagenic *Escherichia coli* isolated from market foods ***

| **Number of Antimicrobials** | **Resistance Pattern** | **Number Observed** |
| --- | --- | --- |
| 6 | AMP-AZI-CPM-CTR-CTX-CTZ | 20 |
| 7 | AMP-AZI-CIP-CPM-CTR-CTX-CTZ | 17 |
| 8 | AMP-AZI-CIP-CPM-CTX-CTZ-IMI-MEM  AMP-AZI-CIP-CPM-CTR-CTX-CTZ-CXT | 12  6 |

***Note**: Only resistance patterns observed in five or more isolates with similar resistance profiles are presented. For animal isolates, no resistance pattern met this threshold.
