# Supplementary material for: One Health surveillance of multidrug-resistant diarrheagenic Escherichia coli in Northeast India
Source: Front Microbiol. 2025 Oct 13;16:1667425. doi: 10.3389/fmicb.2025.1667425 (PMC12554735; doi:10.3389/fmicb.2025.1667425)
Supplement: Supplementary file 4 [file Table_4.docx]

***Supplementary Material***

**Table S4. Class-wise resistance patterns of XDR diarrheagenic *Escherichia coli* isolated from hospital surveillance**

| **Pathotype** | **No. of XDR isolates** | **Resistance Pattern** |
| --- | --- | --- |
| **EPEC** | 1 | PEN, MAC, CEPH-3rd, CEPH-4th, CEPH-MYC, FQ, QN, AG, TET, CBP, PHN |
| **EPEC** | 6 | PEN, MAC, CEPH-3rd, CEPH-4th, CEPH-MYC, FQ, QN, AG, TET, CBP/PHN |
| **EPEC** | 1 | PEN, MAC, CEPH-3rd, CEPH-4th, CEPH-MYC, FQ, QN, AG, TET, FPI |
| **EPEC** | 1 | PEN, MAC, CEPH-3rd, CEPH-4th, CEPH-MYC, FQ, QN, AG, CBP, PHN |
| **ETEC** | 1 | MAC, CEPH-3rd, CEPH-4th, CEPH-MYC, FQ, QN, AG, TET, CBP, PHN |
| **EAEC** | 2 | PEN, MAC, CEPH-3rd, CEPH-4th, CEPH-MYC, FQ, QN, AG, TET, CBP ± PHN |

PEN = Penicillins, MAC = Macrolides, CEPH-3rd = 3rd generation cephalosporins, CEPH-4th = 4th generation cephalosporins, CEPH-MYC = Cephalomycins, FQ = Fluoroquinolones, QN = Quinolones, AG = Aminoglycosides, TET = Tetracyclines, CBP = Carbapenems, PHN = Phenicols, FPI = Folate pathway inhibitors.
