# Supplementary material for: One Health surveillance of multidrug-resistant diarrheagenic Escherichia coli in Northeast India
Source: Front Microbiol. 2025 Oct 13;16:1667425. doi: 10.3389/fmicb.2025.1667425 (PMC12554735; doi:10.3389/fmicb.2025.1667425)
Supplement: Supplementary file 6 [file Table_6.docx]

***Supplementary Material***

**Table S6. Class-wise resistance patterns of XDR diarrheagenic *Escherichia coli* isolated from animals**

| **Pathotype** | **No. of XDR isolates** | **Resistance Pattern** |
| --- | --- | --- |
| EPEC | 2 | PEN, MAC, CEPH-3rd, FQ, QN, TET, CBP, PHN, CEPH-MYC/AG |
| EAEC | 4 | PEN, MAC, CEPH-3rd, CEPH-4th, FQ, QN, AG, TET, CBP, PHN, FPI, ±CEPH-MYC |

PEN = Penicillins, MAC = Macrolides, CEPH-3rd = 3rd generation cephalosporins, CEPH-4th = 4th generation cephalosporins, CEPH-MYC = Cephalomycins, FQ = Fluoroquinolones, QN = Quinolones, AG = Aminoglycosides, TET = Tetracyclines, CBP = Carbapenems, PHN = Phenicols, FPI = Folate pathway inhibitors.
