# Supplementary material for: One Health surveillance of multidrug-resistant diarrheagenic Escherichia coli in Northeast India
Source: Front Microbiol. 2025 Oct 13;16:1667425. doi: 10.3389/fmicb.2025.1667425 (PMC12554735; doi:10.3389/fmicb.2025.1667425)
Supplement: Supplementary file 7 [file Data_Sheet_1.docx]

***Supplementary Material***

**Figure S1.** **Temporal trends in diarrheagenic E. coli (DEC) pathotypes isolated from a) hospital and b) Market (2021–2024)**

**a)**

**
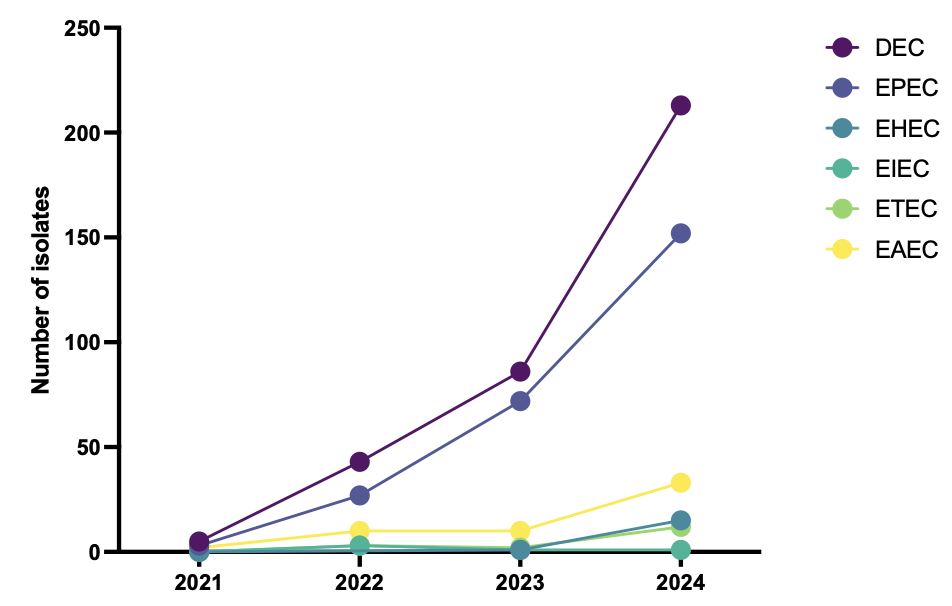
**

**b)**

**
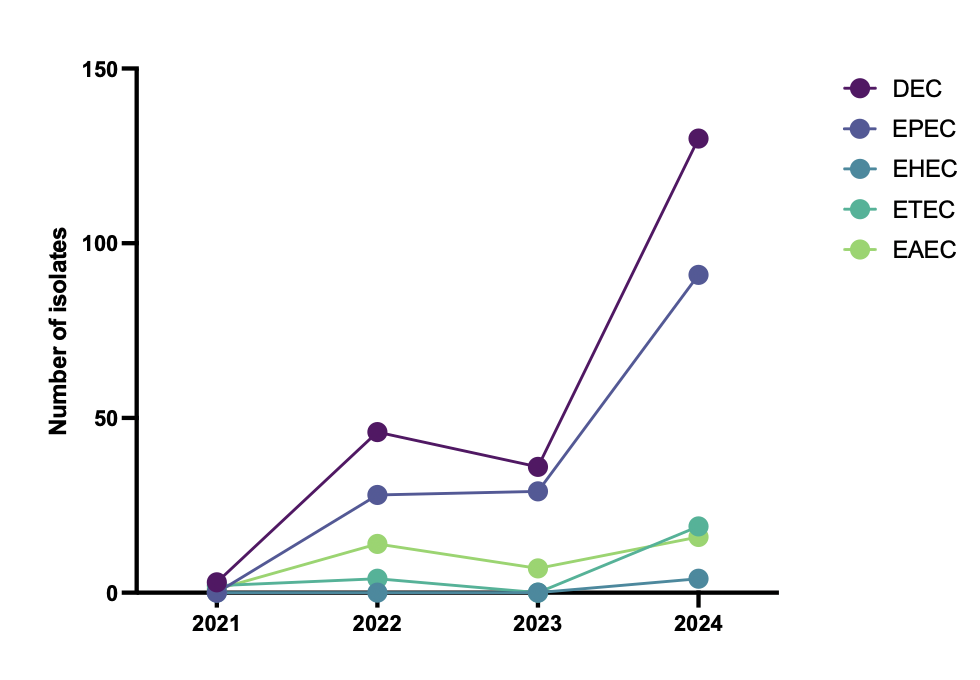
**

***** **Note: 2021 coincided with COVID-19; counts from that year likely underestimate true prevalence.**
